# Microstructure and Mechanical Properties of TiAlMoN Coatings Prepared by Arc Ion Plating

**DOI:** 10.3390/nano16191205

**Published:** 2026-09-24

**Authors:** Haijuan Mei, Rui Wang, Libin Gan, Lixia Cheng, Youqu Shen, Jianming Deng, Kai Li, Qingfeng Zhu, Junfeng Zhao, Dongsen Geng

**Affiliations:** 1 Guangdong Provincial Key Laboratory of Electronic Functional Materials and Devices, Huizhou University, Huizhou 516007, China; 2 Guangxi Key Laboratory of Special Engineering Equipment and Control, Guilin University of Aerospace Technology, Guilin 541004, China; 3 School of Electromechanical Engineering, Guangdong University of Technology, Guangzhou 510006, China

**Keywords:** TiAlMoN, bias voltage, microstructure, mechanical properties

## Abstract

The TiAlMoN coatings were deposited by arc ion plating under different substrate bias voltages, and the effects of substrate bias on the composition, phase structure, microstructure, and mechanical properties of the coatings were systematically investigated. The results showed that, as the bias voltage increased from 0 V to −75 V, enhanced ion bombardment promoted coating densification, the N/(Ti + Al + Mo) atomic ratio decreased from 1.33 to 1.05, and the deposition rate increased from 124.7 nm/min to 138.2 nm/min. XRD peak fitting revealed that the TiAlMoN coatings may consist of three phases: c-TiN, fcc Mo-N, and c-AlN. With increasing negative bias voltage, the preferred (111) orientation became more pronounced, while the Mo-N phase gradually evolved from B1-MoN to γ-Mo_2_N. As the substrate bias voltage increased, the grain size initially reduced and then slightly increased, and the compressive residual stress sharply increased from 0.68 GPa to 2.21 GPa. Owing to coating densification, grain refinement, and increased compressive residual stress, the hardness increased from 16.7 GPa to 33.9 GPa. The adhesion strength reached a maximum of 59.7 N at −25 V and decreased at higher bias voltages because of excessive residual stress. Appropriate control of the substrate bias voltage enables the synergistic optimization of structural strengthening and interfacial stability in TiAlMoN coatings.

## 1. Introduction

Transition-metal nitride coatings are widely used for the surface protection of cutting tools, dies, and mechanical components because of their high hardness, good wear resistance, and chemical stability. As a typical nitride coating, TiN is a well-established hard coating with useful wear resistance [1,2]. However, its overall performance remains insufficient for the increasingly demanding service conditions encountered in high-speed, dry, and high-temperature machining. The incorporation of Al into TiN to form TiAlN coatings further improves hardness, wear resistance, and high-temperature oxidation resistance [3,4,5]. During high-temperature oxidation, the TiAlN coatings form a protective Al-rich oxide layer that suppresses further oxidation, offering considerable potential for high-temperature cutting applications [4,5]. In addition, the Mo-N coatings exhibit high hardness, good chemical stability, and excellent tribological properties [6,7,8]. During friction and oxidation, the Mo-N system can form lubricious Mo-O oxides, thereby reducing the friction coefficient and improving wear resistance [9,10]. Therefore, TiAlN coatings provide distinct advantages in terms of high hardness and high-temperature oxidation resistance, whereas Mo-N coatings offer favorable friction-reduction and wear-resistance characteristics.

To achieve the synergistic optimization of hardness and phase stability, researchers have explored the incorporation of Mo into TiN or TiAlN matrices to form TiMoN or TiAlMoN coatings. Yang et al. deposited TiMoN coatings with different Mo contents by magnetron sputtering and found that Mo alloying increased coating hardness [11]. The variations in Mo content can affect the phase composition, microstructure, and mechanical properties of TiMoN coatings, and an appropriate Mo addition is beneficial for improving coating hardness [12,13]. Further incorporation of Mo into the TiAlN system to form quaternary TiAlMoN coatings has also been reported to enhance the hardness [14,15,16]. Moreover, the addition of Mo can retard the spinodal decomposition of TiAlN and the formation of hexagonal AlN, thereby helping to maintain structural stability at elevated temperatures [17]. In addition to compositional design, substrate bias is a key processing parameter for controlling ion-bombardment energy and film-growth kinetics during coating deposition. An appropriate increase in bias voltage can enhance surface-atom mobility and renucleation, promote coating densification, and improve mechanical properties. However, an excessively high bias voltage may intensify resputtering and compressive residual stress, thereby reducing the adhesion strength or toughness [18,19,20]. In multicomponent TiAlN-based coatings such as TiAlVN, TiAlYN, and TiAlTaN, the bias voltage likewise alters the phase structure, grain size, degree of densification, and residual stress, and consequently affects coating hardness, toughness, and wear resistance [21,22]. Sergevnin et al. prepared the Ti-Al-Mo-N coatings by cathodic arc evaporation and investigated the effects of substrate bias voltage (−120 to −140 V) and nitrogen partial pressure on the microstructure and mechanical properties of the coatings. The results showed that the coatings exhibited a nanocrystalline structure and formed a multilayer architecture consisting of alternating (Ti,Al)N layers and Mo-containing phase layers. By varying the deposition parameters, two types of coatings, (Ti,Al)N-Mo-Mo_2_N and (Ti,Al)N-Mo_2_N, were obtained, among which the (Ti,Al)N-Mo_2_N coating exhibited better mechanical properties [23]. Glatz et al. studied the effects of substrate bias voltage (−40 to −120 V) and Mo content on the mechanical properties of Ti_1−x−y_Al_x_Mo_y_N coatings and found that the hardness reached a maximum of 39.0 GPa at −120 V and was almost independent of Mo content [24]. These studies indicate that substrate bias voltage has a significant influence on the microstructure and mechanical properties of TiAlMoN systems.

However, existing studies have mainly focused on higher bias ranges, and less attention has been paid to high-Al monolithic TiAlMoN coatings deposited in the lower bias range (0 to −75 V). As for the TiAlMoN coatings, variations in bias voltage alter ion bombardment and resputtering and consequently affect the deposition of Ti, Al, Mo, and N, as well as the phase structure, microstructure, and mechanical properties. Therefore, in this study, TiAlMoN coatings were investigated by systematically varying the substrate bias voltage, evaluating the resulting changes in chemical composition, microstructure, residual stress, and mechanical properties, and analyzing the relationships among bias voltage, structure, and properties.

## 2. Experimental Details

### 2.1. Coating Deposition

The TiAlMoN coatings were deposited on single-side-polished cemented carbide, stainless steel, and single-crystal silicon substrates by arc ion plating (AIP). A single Ti_25_Al_65_Mo_10_ alloy target with dimensions of Ø100 mm × 40 mm and a purity of 99.9% was mounted opposite the substrate holder at a target-to-substrate distance of 240 mm; the substrate holder rotated at 5 rpm during deposition. Before deposition, all substrates were ultrasonically cleaned sequentially in acetone and absolute ethanol for 15 min, dried, and mounted on the substrate holder. After the chamber was evacuated to 9.0 × 10^−4^ Pa, the substrates were heated to 200 °C. High-purity Ar was then introduced, the working pressure was adjusted to 1.0 Pa, and glow-discharge cleaning was performed for 15 min at a bias voltage of −800 V to remove surface contaminants and improve coating/substrate interfacial adhesion. High-purity N_2_ was then introduced at a flow rate of 25 sccm, and the total chamber pressure was maintained at 1.0 Pa throughout TiAlMoN deposition by AIP. During deposition, the target current was maintained at 80 A, the substrate bias voltages were set to 0, −25, −50, and −75 V, and the deposition time was fixed at 30 min. The remaining deposition parameters are listed in Table 1.

### 2.2. Coating Characterization

The surface and cross-sectional morphologies of the coatings were characterized by scanning electron microscopy (SEM, Vega3, Tescan, Brno, Czech Republic), and the elemental compositions were determined by EDS. The phase structures were analyzed using an X-ray diffractometer (XRD, MiniFlex 600, Rigaku, Akishima, Japan) with Cu Kα radiation (λ = 1.5406 Å). Overlapping diffraction peaks were deconvoluted using Gaussian functions, and the lattice parameters and crystallite sizes were estimated according to Bragg’s law and the Scherrer equation [25]. Chemical bonding states were analyzed by X-ray photoelectron spectroscopy (XPS, ESCALAB 250Xi, Thermo Fisher Scientific, Waltham, MA, USA) with Al Kα radiation, and all binding energies were calibrated against the C 1s peak at 284.8 eV. Based on the substrate-curvature method [26], the residual stresses in the coatings were measured using a thin-film stress tester (FST-1000, SuPro Instruments, Shenzhen, China). Hardness and elastic modulus were measured by a nanoindenter (NHT^2^, CSM, Peseux, Switzerland) under a maximum load of 25 mN. At least 5 points were measured for each sample, and the maximum indentation depth was maintained below 10% of the coating thickness to minimize the substrate effect. The coating/substrate adhesion strength was evaluated using a high-load scratch tester (RST, CSM, Peseux, Switzerland) with a maximum load of 100 N and a scratch length of 3 mm. Residual-stress and scratch measurements were each repeated three times for every bias condition.

## 3. Results

### 3.1. Microstructure

Table 2 lists the chemical compositions and deposition rates of the TiAlMoN coatings deposited under different substrate bias voltages. As the substrate bias voltage increased from 0 V to −75 V, the Ti, Al, and Mo contents in the coatings increased from 11.3 at.%, 28.2 at.%, and 3.5 at.% to 13.0 at.%, 31.5 at.%, and 4.3 at.%, respectively. Meanwhile, the N content decreased from 57.0 at.% to 51.2 at.%, the coating thickness increased from 3.74 μm to 4.15 μm, and the deposition rate increased from 124.7 nm/min to 138.2 nm/min. These changes indicate that the bias voltage altered the energy of the ions arriving at the growing surface and their effective deposition probability. In typical PVD processes, as the negative bias voltage increases, the deposition rate generally first increases and then decreases. This is because at low bias voltages, enhanced ion attraction is dominant, whereas beyond a certain threshold bias, resputtering gradually becomes dominant, leading to a decrease in deposition rate. Similar phenomena have been reported in TiAlN coatings; excessive bias voltage intensifies resputtering and thereby reduces the deposition rate [19]. In this study, because the bias voltage (0 to −75 V) is relatively low, the deposition rate first increased and then tended to stabilize, indicating that enhanced ion attraction and surface migration remained dominant within this bias voltage range. In addition, stronger ion bombardment of the growing surface with increasing negative bias caused the N content to decrease continuously, and the N/(Ti + Al + Mo) atomic ratio reduced from 1.33 to 1.05. This result can be attributed to the resputtering and desorption of weakly bonded N atoms by energetic species, accompanied by a relative increase in the effective deposition fraction of metal ions. Similar bias-induced resputtering and compositional changes have been reported for TiAlN coatings [19,20]. After normalization of the metal contents, the coating composition changed from Ti_26.3_Al_65.6_Mo_8.1_N to Ti_26.6_Al_64.6_Mo_8.8_N, which was close to the metallic ratio of the Ti_25_Al_65_Mo_10_ target. This result indicates that no significant selective loss occurred among Ti, Al, and Mo and that the principal effect of the bias voltage on chemical composition was the adjustment of the N content and overall stoichiometry.

Figure 1 shows the surface SEM morphologies of the TiAlMoN coatings deposited under different substrate bias voltages. As shown in Figure 1a, without an applied bias, numerous spherical particles of nonuniform size and local pores were distributed over the coating surface. These features are typical droplet surface defects generated during arc ion plating. As cathodic arc spots move rapidly across the target surface, localized melting occurs, and some micrometer-scale metal droplets reach the substrate with the plasma before being fully ionized. These droplets are subsequently encapsulated by the deposited nitride layer, forming protruding particles. Local pores may form when some droplets detach during deposition or when geometric shadowing prevents subsequent depositing species from completely filling the regions around the droplets. When the negative bias increased, the number of observable particles on the coating surface gradually increased, and the surface relief became more pronounced, indicating that the bias voltage affected the surface evolution of droplet-related defects. This phenomenon is mainly associated with the enhanced ion bombardment and resputtering effect at higher bias voltages. With increasing negative bias, the energy of the ions arriving at the substrate surface increased, subjecting the continuous regions to more intense ion bombardment and resputtering. The more energetic incident ions altered the balance between deposition and removal around the droplets, exposing some droplet particles more readily, thereby accentuating the surface-particle features and roughness. Pfeiler et al. also found that substrate bias voltages significantly affected droplet density and droplet-induced surface roughness in arc-evaporated Ti-Al-V-N coatings, thereby controlling the coating’s surface morphology [21].

Figure 2 shows cross-sectional SEM morphologies of TiAlMoN coatings deposited under different substrate bias voltages. As shown in Figure 2a, the coating deposited without an applied bias exhibited a pronounced columnar structure with longitudinal gaps and local pores between the columns. This morphology was mainly attributable to the low energy of the depositing species and the limited surface mobility of adatoms under the unbiased condition, which resulted in a pronounced shadowing effect during growth and produced a relatively porous columnar structure. However, as the negative bias increased, the gaps between the columns gradually decreased. At bias voltages of −50 V and −75 V, the coating cross sections became distinctly more continuous and dense, as shown in Figure 2c,d. This was primarily attributed to the enhanced surface migration and rearrangement of depositing atoms caused by the increased ion-bombardment energy, together with enhanced defect filling and renucleation, which suppressed intercolumnar pores. The study by Baptista et al. on TiAlYN and TiAlTaN coatings likewise showed that increasing the substrate bias enhanced the mobility of depositing species, promoted microstructural densification, and was accompanied by grain refinement [22]. Pfeiler et al. also noted that enhanced ion bombardment disrupted continuous local crystal growth and promoted renucleation, thereby altering the competitive growth of the columns [21]. The cross-sectional morphologies further show that, under all bias voltage conditions, the columns extended from the substrate/coating interface toward the surface along the coating-thickness direction, indicating that growth was approximately perpendicular to the substrate surface. The delaminated regions in the micrographs were mainly produced by brittle fracture along the column boundaries during cross-sectional fracture preparation. This observation indicates that column boundaries are relatively susceptible to crack propagation during fracture and further confirms the pronounced columnar growth of the coatings. As the negative bias increased, the intercolumnar pores gradually decreased, and the boundaries became denser, demonstrating that enhanced ion bombardment improved bonding between adjacent columns and increased the overall coating density.

Figure 3 presents the XRD patterns, peak-deconvolution results, lattice parameters, and grain sizes of the coatings deposited under different substrate bias voltages. In Figure 3a, without an applied bias, pronounced nitride diffraction peaks appeared near 36.4° and 43.7°, corresponding to the (111) and (200) planes of the face-centered cubic (fcc) structure, respectively. Weak t-Al_5_Ti_3_(441) and t-Mo_9_Ti_4_(511) diffraction peaks were also observed near 51.2° and 56.9°, indicating the presence of small amounts of intermetallic compounds in addition to the nitride phases. As the bias voltage increased to −25 V, the intensities of the nitride diffraction peaks decreased, while the peaks associated with the intermetallic compounds weakened markedly, indicating that enhanced ion bombardment and surface migration facilitated the reactive deposition process. When the bias voltage further increased to −50 V and −75 V, the (111) diffraction peak intensified significantly, and a weak (220) diffraction peak appeared near 62.4°, indicating that a pronounced (111) preferred orientation gradually developed in the coatings. The increased energy of the incident ions at higher bias voltages enhanced adatom surface mobility and altered the competitive growth of different crystallographic planes, thereby promoting preferential growth along the (111) orientation [18,20]. Because the (111) diffraction peak in the 35 ~ 39° range exhibited a distinctly asymmetric profile, Gaussian functions were used to deconvolute the peak and resolve its components [27]. As shown in Figure 3b,c, the asymmetric (111) peak could be deconvoluted into three subpeaks corresponding to c-TiN, fcc Mo-N, and c-AlN, suggesting that these three phases may coexist in the TiAlMoN coatings prepared in this study. Previous studies have reported that TiAlMoN coatings can also form a single TiN-based fcc solid-solution phase, in which Al and Mo atoms substitutionally dissolve in the TiN lattice [14,15]. For the middle-Al Ti_45_Al_45_Mo_10_ target, single-phase cubic TiAlMoN formed in the Ti_1−x−y_Al_x_Mo_y_N coatings [24]. In contrast, the high-Al Ti_25_Al_65_Mo_10_ target and the strongly nonequilibrium arc-ion-plating conditions used in this study may have hindered the formation of a completely homogeneous solid solution, thereby promoting the coexistence of the c-TiN, fcc Mo-N, and c-AlN nitride phases. As the negative bias increased, the overall (111) diffraction peak shifted toward higher angles, indicating a gradual decrease in the corresponding interplanar spacing. As shown in Figure 3d, when the substrate bias voltage increased from 0 V to −25 V, the lattice parameter of the Mo-N phase decreased slightly from 4.207 Å to 4.204 Å. When the bias voltage was further increased to −50 V and −75 V, the lattice parameter decreased to 4.175 Å and 4.170 Å, respectively. According to the literature [28,29,30], fcc Mo-N comprises the B1-MoN and γ-Mo_2_N phases, with corresponding lattice parameters of 4.20 ~ 4.27 Å and 4.16 ~ 4.19 Å, respectively. Thus, with increasing bias voltage, the Mo-N phase gradually evolved from B1-MoN to γ-Mo_2_N. This structural evolution corresponded to the reduction in N content shown in Table 2. The enhanced resputtering and desorption of N atoms at higher bias voltages caused the Mo-N system to evolve toward a relatively Mo-rich structure. The grain sizes in Figure 3e were calculated using the Scherrer equation [25]. The average grain size of the three nitride phases gradually decreased from 18.5 nm to 13.8 nm at −50 V and then increased slightly to 14.2 nm at −75 V. At moderate bias voltages, energetic-particle bombardment increased the number of nucleation sites and restricted continuous grain growth, resulting in grain refinement. At a still higher bias voltage, enhanced surface diffusion and local heat input favored grain rearrangement and growth, producing the observed competition between initial refinement and subsequent slight coarsening.

Figure 4 shows the fitted XPS spectra of the TiAlMoN coatings. The Ti 2p spectrum in Figure 4a can be deconvoluted into two groups of chemical states corresponding to Ti-N and TiO_x_. The Ti-N-related peaks are located at approximately 454.3 eV and 460.0 eV, whereas the TiO_x_ peaks are located at approximately 456.4 eV and 462.1 eV [4]. The oxide peaks mainly originate from spontaneous surface oxidation and reactions with adsorbed oxygen after exposure of the samples to air. Such Ti-O signals are common in XPS analyses of nitride coatings [31,32]. After fitting, the Al 2p spectrum in Figure 4b can be resolved into two subpeaks, corresponding to the Al-N peak at 73.9 eV and the Al_2_O_3_ peak at 74.9 eV [33]. In Figure 4c, the asymmetric Mo 3d spectrum can also be deconvoluted into two groups of peaks corresponding to Mo-N (228.2 eV, 231.4 eV) and MoO_2_ (229.5 eV, 232.7 eV) [34]. This result indicates that Mo was mainly present in a nitride-bonding environment in the coatings, while a small amount of surface oxidation was also present. The N 1s spectrum in Figure 4d can be deconvoluted into three subpeaks at approximately 396.3 eV, 396.9 eV, and 397.6 eV, which are assigned to Mo-N, Ti-N, and Al-N bonds, respectively [13]. These XPS results support the presence of local Ti-N, Mo-N, and Al-N bonding environments. As the negative bias voltage increased, the Ti 2p, Al 2p, Mo 3d, and N 1s spectra shifted slightly toward lower binding energies, while the principal peak shapes and chemical components remained unchanged. This indicates that the bias voltage did not alter the primary bonding types in the TiAlMoN coatings but modified the local electronic structure. In conjunction with the decrease in N/(Ti + Al + Mo) shown in Table 2 and the changes in lattice parameter shown in Figure 3, these shifts can be attributed to changes in the local coordination and charge-transfer states around the metal atoms caused by enhanced ion bombardment and N loss.

### 3.2. Mechanical Properties

Figure 5 shows the variation in residual stress in the TiAlMoN coatings deposited under different substrate bias voltages. All coatings exhibited compressive residual stress, and the absolute value gradually increased from 0.68 GPa to 2.21 GPa with increasing negative bias voltage. The coating deposited without an applied bias exhibited the lowest compressive residual stress, consistent with the relatively porous columnar structure. The larger intercolumnar gaps and local pores provided space for partial relaxation of the stress generated during deposition. As the bias voltage increased, the kinetic energy of the incident ions increased, and intensified ion bombardment caused a greater number of energetic particles to interact with the growing surface. This interaction induced atomic peening, point-defect formation, and local lattice distortion while promoting coating densification, thereby causing a continuous increase in compressive residual stress. An increase in compressive residual stress with increasing negative bias voltage has also been observed in the TiAlN coatings deposited by arc ion plating and was attributed to the accumulation of defects and lattice distortion induced by energetic-ion bombardment [18]. Pfeiler et al. further noted that the defects are generated and annihilated simultaneously during ion bombardment. In the low-to-moderate bias range, defect generation dominates, and the compressive residual stress increases with the bias voltage. At higher energies, enhanced atomic mobility may promote defect annihilation, causing the increase in stress to level off or even reverse [21]. The maximum bias voltage used in this study was only −75 V, and the compressive residual stress increased monotonically within this range, indicating that the defect accumulation and microstructural densification induced by ion bombardment remained dominant. The shift in the diffraction peaks toward higher angles and the lattice contraction shown in Figure 3 are also consistent with this stress evolution.

Figure 6 presents the nanoindentation loading-unloading curves, hardness, elastic modulus, and H^3^/E^*2^ ratios of the TiAlMoN coatings deposited under different substrate bias voltages. As shown in Figure 6a, the maximum indentation depths of all samples were below 300 nm and less than 10% of the coating thickness, effectively minimizing the substrate effect. As the negative bias increased, the maximum indentation depth gradually decreased, indicating enhanced resistance to indentation deformation. As shown in Figure 6b, the coating deposited without an applied bias exhibited a relatively low hardness of 16.7 GPa and an elastic modulus of 291.1 GPa. This was mainly associated with the porous columnar structure and numerous intercolumnar pores shown in Figure 2, while the small amount of intermetallic compounds shown in Figure 3 may also have contributed. With increasing negative bias voltage, the hardness and elastic modulus gradually increased. At −75 V, the hardness and elastic modulus reached 33.9 GPa and 493.9 GPa, respectively. This improvement was primarily attributable to the synergistic strengthening effects of microstructural densification, grain refinement, and increased compressive residual stress. As the negative bias voltage increased, enhanced ion bombardment promoted the surface migration and rearrangement of depositing atoms, reduced intercolumnar pores, and increased the coating density. Meanwhile, grain refinement increased the number of grain boundaries, which impeded dislocation motion and produced a fine-grain strengthening effect. Similar bias-induced strengthening through densification and grain refinement has been reported for TiAlN and other multicomponent TiAlN-based coatings [18,20,21,22]. Sergevnin et al. [23] prepared (Ti,Al)N-Mo_2_N multilayer coatings by adjusting the bias voltage and nitrogen partial pressure and reported a hardness of up to 40 GPa. Glatz et al. [24] used (Ti_0.5_Al_0.5_)_1−y_Mo_y_ targets to prepare TiAlMoN coatings and reported a hardness of 36.6 GPa at −80 V, which is also higher than that of the present study at comparable bias voltages. These differences can be mainly attributed to the differences in coating architecture (multilayer vs. monolithic), Al content (high-Al vs. medium-Al), Mo content, and deposition system. In addition, the increase in compressive residual stress with increasing bias enhanced the resistance of the coatings to localized plastic deformation and contributed to the increase in hardness. The H^3^/E*^2^ ratio is commonly used to evaluate the resistance of hard coatings to plastic deformation [35]. With increasing bias voltage, the H^3^/E*^2^ ratio gradually increased from 0.046 to 0.133, indicating that the coating deposited at a high bias exhibited greater resistance to plastic deformation.

Figure 7 shows the scratch morphologies and critical loads of the TiAlMoN coatings deposited under different substrate bias voltages. As shown in Figure 7a, all coatings exhibited cracks and local delamination to varying degrees under a maximum linearly increasing load of 100 N. Based on the appearance of semicircular coating cracks within the scratch track (L_C1_), spallation of the coating along the track edges (L_C2_), initial coating failure (L_C3_), and complete exposure of the substrate (L_C4_), the failure process can be divided into four critical stages. The critical load L_C2_ is commonly used as the principal indicator of coating/substrate adhesion strength [36]. As shown in Figure 7b, the coating deposited without an applied bias exhibited an adhesion strength of only 13.2 N, mainly because of its relatively porous columnar structure and numerous intercolumnar defects, along which cracks readily initiated and propagated. When the bias voltage was increased to −25 V, the adhesion strength increased markedly to 59.7 N. Moderate ion bombardment promoted atomic migration and coating densification, reduced intercolumnar pores and structural defects, improved load transfer, and limited crack initiation and propagation. When the bias voltage was further increased to −50 V and −75 V, the adhesion strength decreased to 26.5 N and 22.8 N, respectively. As the negative bias voltage increased, the compressive residual stress in the coatings increased markedly. Excessive compressive stress increases the elastic energy stored within the coating and more readily induces crack propagation and local delamination under scratch loading, thereby reducing the adhesion strength. Similarly, although a high bias voltage is beneficial for increasing the hardness, excessively intense ion bombardment causes stress accumulation and increases the elastic energy, thereby reducing the adhesion strength and toughness [18]. Cao et al. also observed that the adhesion strength of TiAlN coatings initially increased and then decreased with increasing bias voltage, indicating that a moderate negative bias provides a better balance between microstructural densification and interfacial stability [20].

## 4. Conclusions

TiAlMoN coatings were deposited by arc ion plating under different substrate bias voltages, and the relationships between substrate bias voltage, structural evolution, and mechanical properties were systematically analyzed. As the negative bias increased, enhanced ion bombardment promoted the migration and rearrangement of depositing atoms, transforming the coating from a porous columnar structure into a denser columnar structure while decreasing the N content and increasing the deposition rate. XRD peak fitting suggested that the coatings may contain coexisting c-TiN, fcc Mo-N, and c-AlN phases. When the bias voltage increased, preferential growth along the (111) orientation became more pronounced; the grain size initially decreased from 18.5 nm to 13.8 nm and then increased slightly to 14.2 nm, and the Mo-N phase gradually evolved from B1-MoN to a γ-Mo_2_N-type structure. As the bias voltage increased, the compressive residual stress increased from 0.68 GPa to 2.21 GPa, while the hardness and elastic modulus increased from 16.7 GPa and 291.1 GPa to 33.9 GPa and 493.9 GPa, respectively. The adhesion strength of the coatings initially increased and then decreased, reaching a maximum of 59.7 N at −25 V. Increasing the bias voltage enhanced ion bombardment, which promoted microstructural densification, grain refinement, and increased compressive residual stress, thereby improving hardness and resistance to plastic deformation. However, excessive residual stress reduced the coating adhesion strength. Thus, in this study, −25 V represents the most favorable condition for maximizing adhesion strength, whereas −75 V produces the greatest hardness and resistance to plastic deformation.

## Figures and Tables

**Figure 1:**
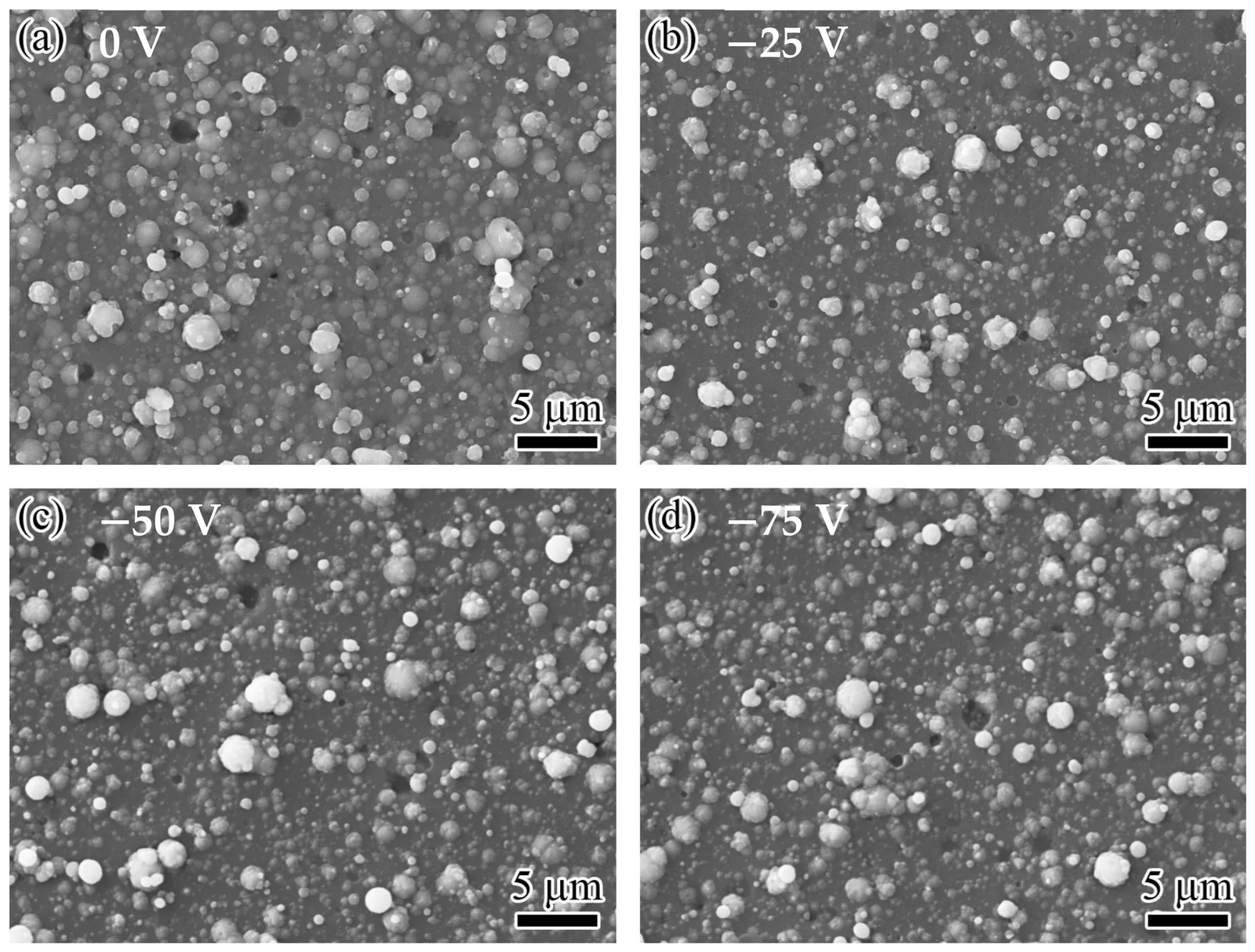
Surface micrographs of the TiAlMoN coatings at various bias voltages: (**a**) 0 V, (**b**) −25 V, (**c**) −50 V, and (**d**) −75 V.

**Figure 2:**
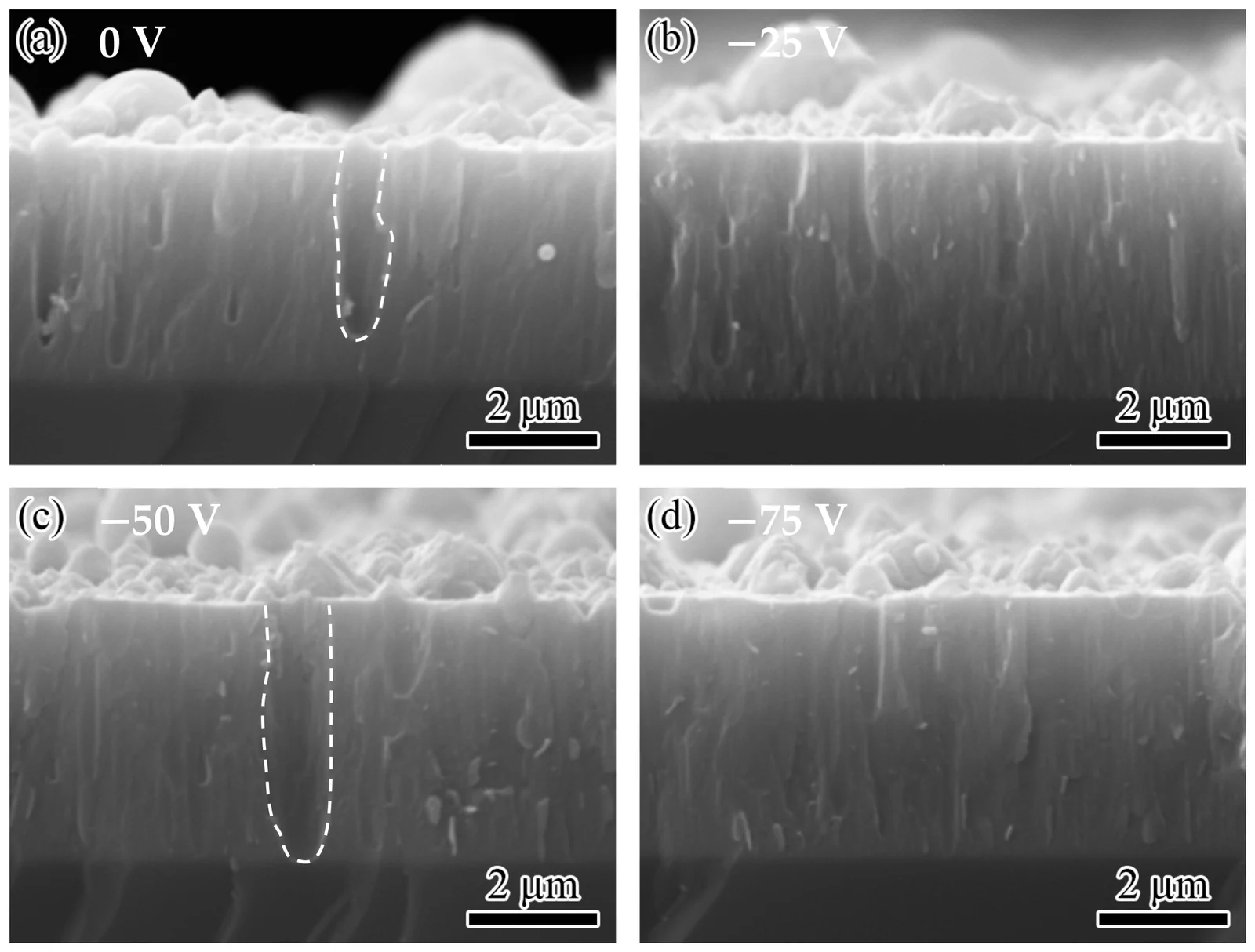
Cross-sectional micrographs of the TiAlMoN coatings at various bias voltages: (**a**) 0 V, (**b**) −25 V, (**c**) −50 V, and (**d**) −75 V.

**Figure 3:**
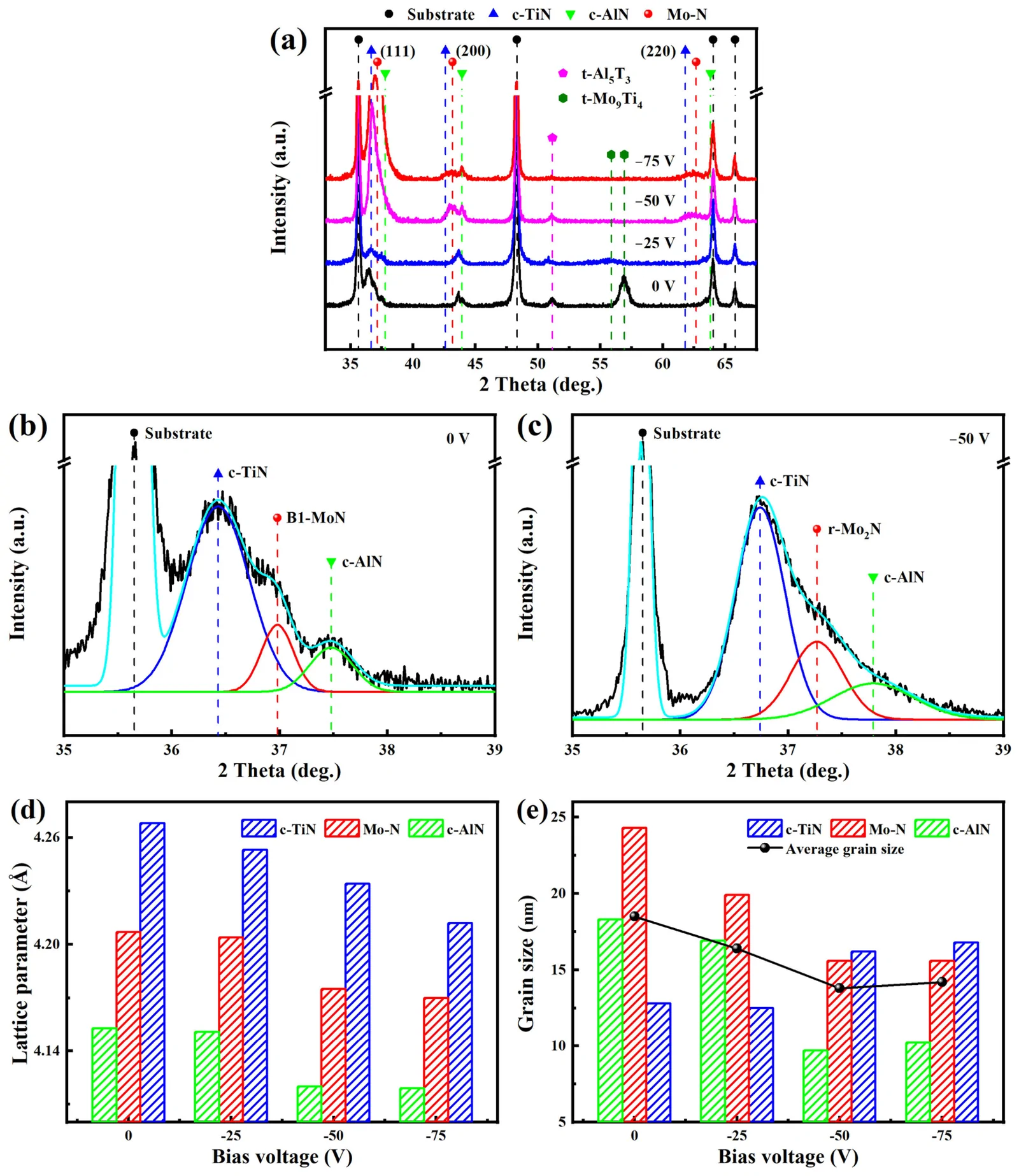
XRD pattern (**a**), (111) fitting peak at 0 V (**b**), (111) fitting peak at −50 V (**c**), lattice parameter (**d**), and grain size (**e**) of the TiAlMoN coatings at various bias voltages.

**Figure 4:**
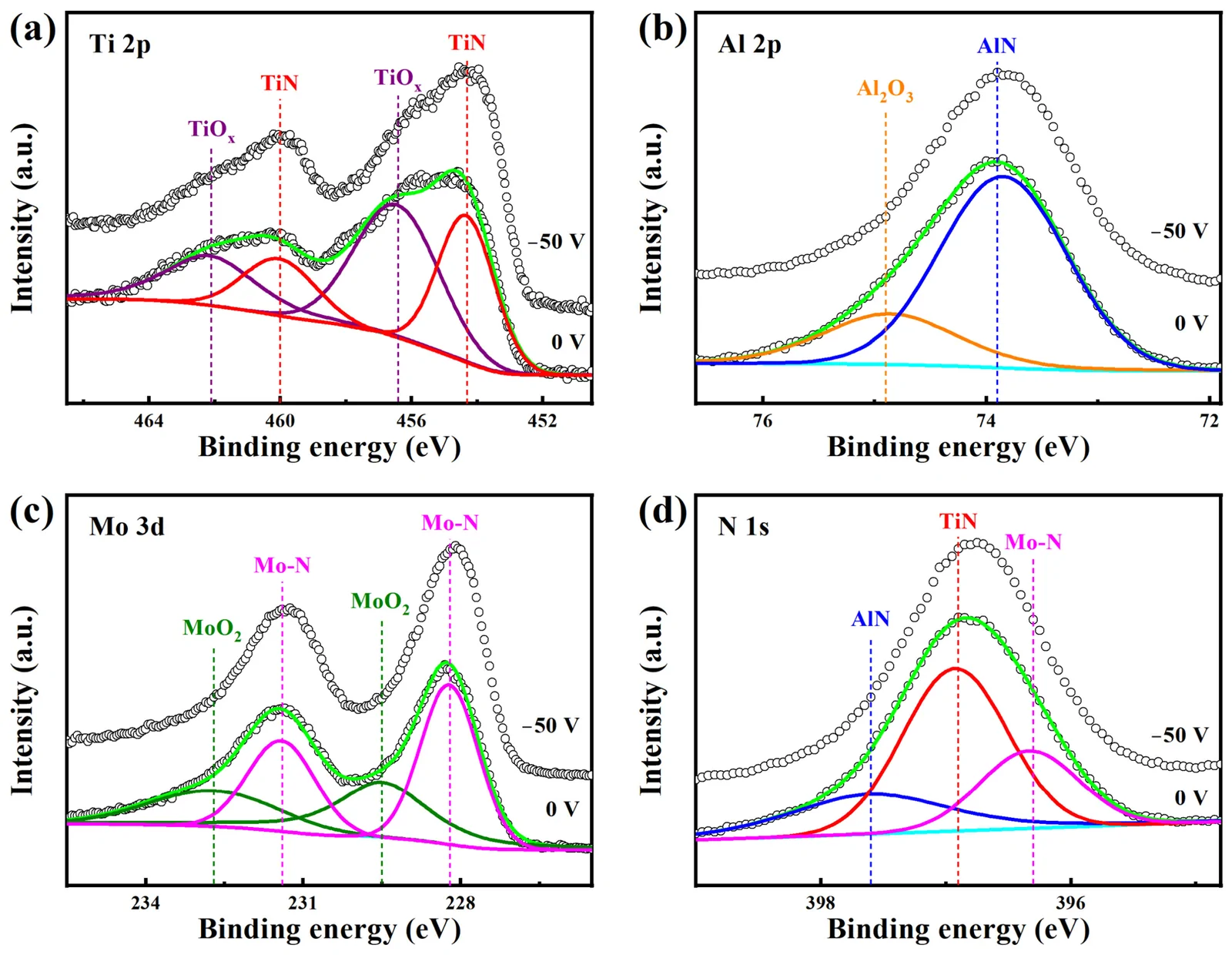
Fitted XPS spectra of the TiAlMoN coatings at various bias voltages: (**a**) Ti 2p, (**b**) Al 2p, (**c**) Mo 3d, and (**d**) N 1s.

**Figure 5:**
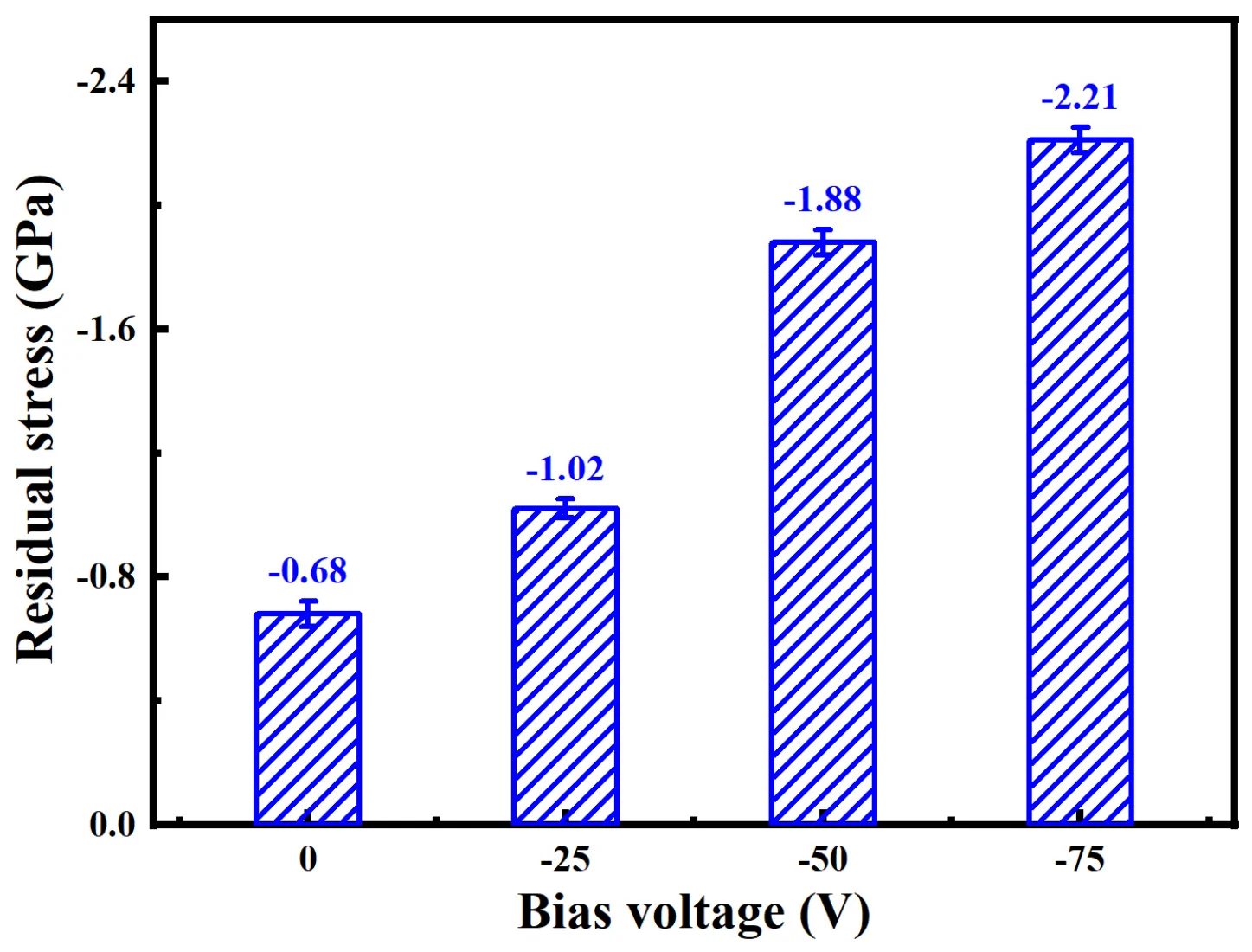
Residual stress of the TiAlMoN coatings at various bias voltages.

**Figure 6:**
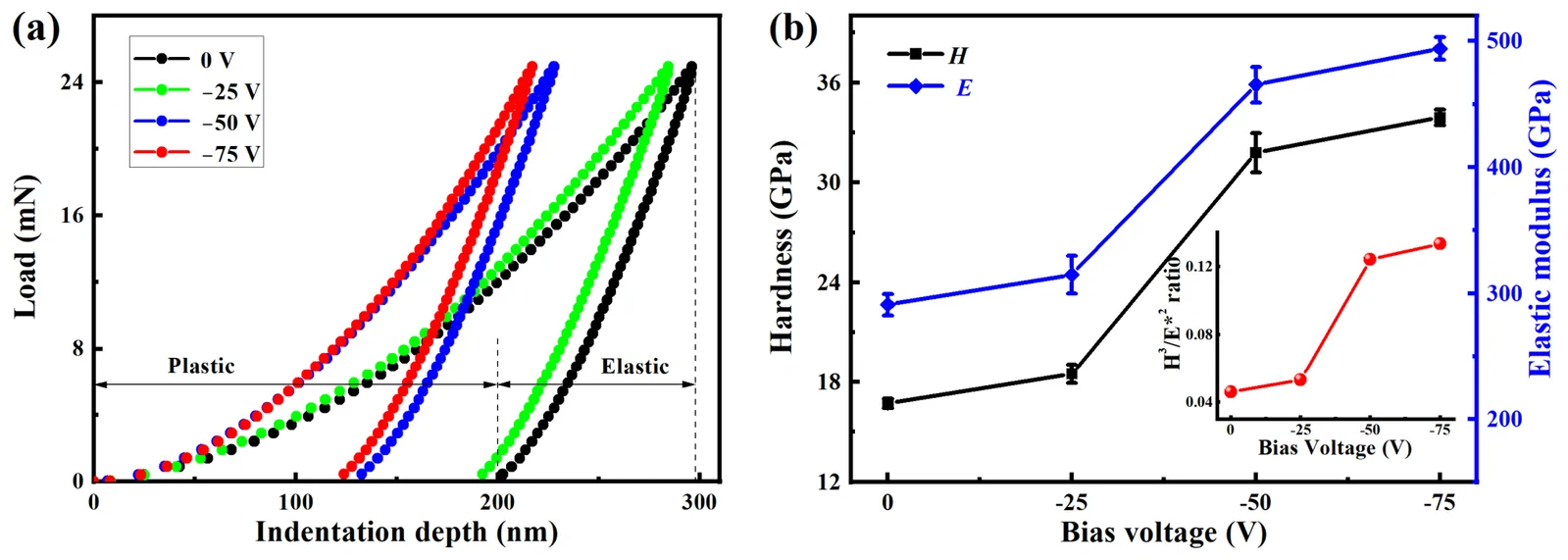
Indentation load-displacement curves (**a**), hardness, elastic modulus, and H^3^/E*^2^ ratio (**b**) of the TiAlMoN coatings at various bias voltages.

**Figure 7:**
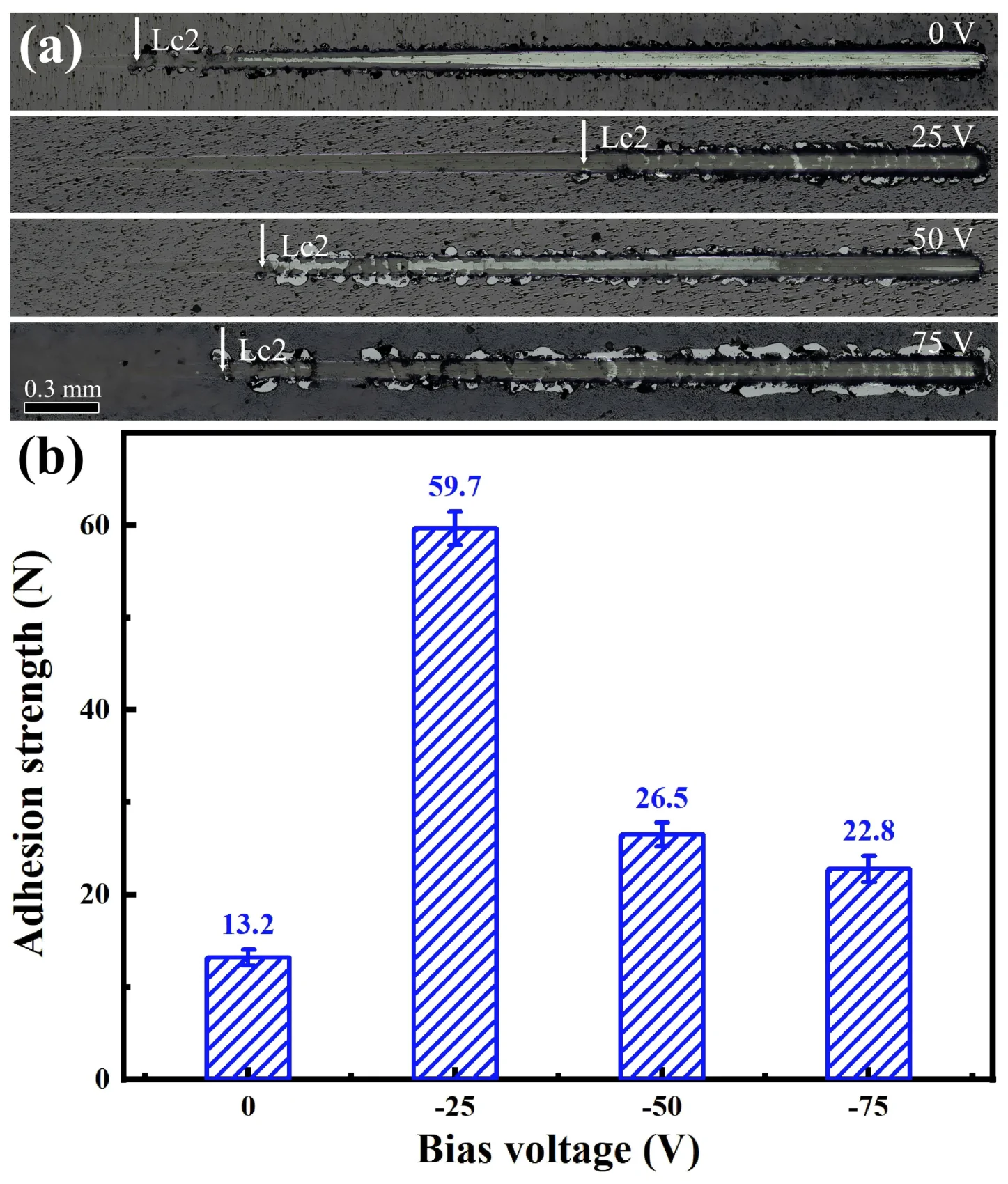
Scratch micrographs (**a**) and adhesion strength (**b**) of the TiAlMoN coatings at various bias voltages.

**Table 1:** Deposition parameters of the TiAlMoN coatings.

Parameters	TiAlMoN
Base pressure (Pa)	9.0 × 10^−4^
Working temperature (°C)	200
Working pressure (Pa)	1.0
N_2_ flow rate (sccm)	25
Target-to-substrate distance (mm)	240
Target material (at.%)	Ti_25_Al_65_Mo_10_
Target current (A)	80
Substrate bias voltage (V)	0, −25, −50, −75
Deposition time (min)	30

**Table 2:** Chemical composition, thickness, and deposition rate of TiAlMoN coatings.

Bias Voltage (V)	Chemical Composition (at.%)				N/(Ti + Al + Mo) Ratio	Thickness (μm)	Deposition Rate (nm/min)
	Ti	Al	Mo	N			
0	11.3	28.2	3.5	57.0	1.33	3.74	124.7
−25	12.3	30.5	4.1	53.1	1.13	4.08	135.8
−50	12.6	30.7	4.2	52.5	1.11	4.11	136.8
−75	13.0	31.5	4.3	51.2	1.05	4.15	138.2

## Data Availability

The original contributions presented in this study are included in the article. Further inquiries can be directed to the corresponding authors.
